# In vivo interactome profiling by enzyme‐catalyzed proximity labeling

**DOI:** 10.1186/s13578-021-00542-3

**Published:** 2021-01-29

**Authors:** Yangfan Xu, Xianqun Fan, Yang Hu

**Affiliations:** 1 Department of Ophthalmology, Stanford University School of Medicine, Palo Alto, CA 94304 USA; 2 Department of Ophthalmology, Ninth People’s Hospital, Shanghai JiaoTong University School of Medicine, Shanghai, People’s Republic of China; 3 Shanghai Key Laboratory of Orbital Diseases and Ocular Oncology, Shanghai, People’s Republic of China

**Keywords:** Proximity labeling, Protein interactome, APEX, BioID, TurboID

## Abstract

Enzyme-catalyzed proximity labeling (PL) combined with mass spectrometry (MS) has emerged as a revolutionary approach to reveal the protein-protein interaction networks, dissect complex biological processes, and characterize the subcellular proteome in a more physiological setting than before. The enzymatic tags are being upgraded to improve temporal and spatial resolution and obtain faster catalytic dynamics and higher catalytic efficiency. In vivo application of PL integrated with other state of the art techniques has recently been adapted in live animals and plants, allowing questions to be addressed that were previously inaccessible. It is timely to summarize the current state of PL-dependent interactome studies and their potential applications. We will focus on in vivo uses of newer versions of PL and highlight critical considerations for successful in vivo PL experiments that will provide novel insights into the protein interactome in the context of human diseases.

## Background

Proteins generally form complexes, organelles or other assemblies and create interacting networks that are essential for cellular structure and functional integrity. The execution of biological function and progression of human disease are intimately tied to protein-protein interactions (PPIs), which are characterized by proximity, affinity and duration [1]. Exploration of PPIs underlying intricate cellular signaling and regulatory mechanisms has required efforts to circumvent the technical defects in many of the traditional approaches. The interactome mapping methods commonly employed are affinity purification-mass spectrometry (AP-MS) and yeast two-hybridization [2, 3]. However, these methods very often fail to reveal in vivo PPIs because they require ex vivo manipulation and subcellular fractionation for enrichment; these processes are associated with low validation rates and high false positive rates [4, 5]. Utilization of AP-based methods requires that cells first being lysed to release the bait protein for purification, which poses significant challenges to faithfully preserving its in vivo interacting status. Yeast two-hybridization assay can only suggest the potential interaction of two proteins, rather than the interaction that actually takes place in vivo [5]. Fluorescence-based techniques are most suitable for validating the candidate interacting partners of a given protein, particularly in such applications as a high-throughput drug screening platform, rather than for identifying novel partners [6, 7]. The lack of effective tools to acquire accurate information about protein distribution, protein partners, and protein complex composition remains a major challenge in these fields [8].

In the past decade, enzyme-catalyzed proximity labeling (PL) has developed as a novel alternative method to label and capture not only the proteins that interact directly with the protein of interest (POI), but also the proteins in proximity to the POI [9–11]. In a PL system, a promiscuous labeling enzyme is fused in frame to the POI or subcellular compartment marker proteins in living cells. Enzymatic catalyzation will covert an inert substrate into a reactive but short-lived intermediate, which will then covalently label the nearby biomolecules (proteins, RNA and DNA) in a promiscuous and proximity-dependent manner. Because in most cases the small-molecule substrate for labeling contains biotin moiety, the biotinylated proteins can be selectively enriched by affinity purification using neutravidin or streptavidin coated magnetic or agarose beads. Streptavidin shows a stronger intrinsic binding affinity toward biotin and a lower nonspecific binding than neutravidin [12, 13]. Due to higher reproducibility, purity-specificity and ease of use, magnetic beads have become the preferred support for small-scale experiments, whereas agarose beads are more economical when large amounts of purified targeting biomolecules are required. Background contamination is likely to be small because avidin-biotin interaction can withstand harsh and stringent purification conditions and the endogenous biotinylation is relatively low in mammalian cells [11]. The purified proteins are subsequently identified by high-throughput liquid chromatography coupled to mass spectrometry (LC-MS/MS). Another superior feature of PL is that it can capture transient or weak interactions that are often overlooked by conventional AP approaches. Therefore, this technique facilitates the sensitive, specific and timely detection of the interactome of a POI in vivo, which is critical for understanding its broad molecular functions quickly, simply and reliably. Accumulating publications have broadened the range of the bait proteins, from nuclear membrane proteins and transcriptional factors to ubiquitin ligases [9, 14, 15], and, recently, even to RNA-protein interactions [16–20].

In addition to its extensive use in cultured mammalian cells [9, 14, 21, 22], PL has been rapidly adapted for in vivo application in a wide variety of research projects and models, including yeast [23, 24], plant protoplasts [25, 26], parasites [27–29], mouse [30, 31], flies and worms [32]. In this review, we will focus on the evolution of powerful PL approaches and the important considerations for PL experimental design, especially the in vivo utilization of PL methods integrated with other sophisticated approaches to profile protein interactome with high confidence.

### The APEX and HRP system

**Fig. 1:**
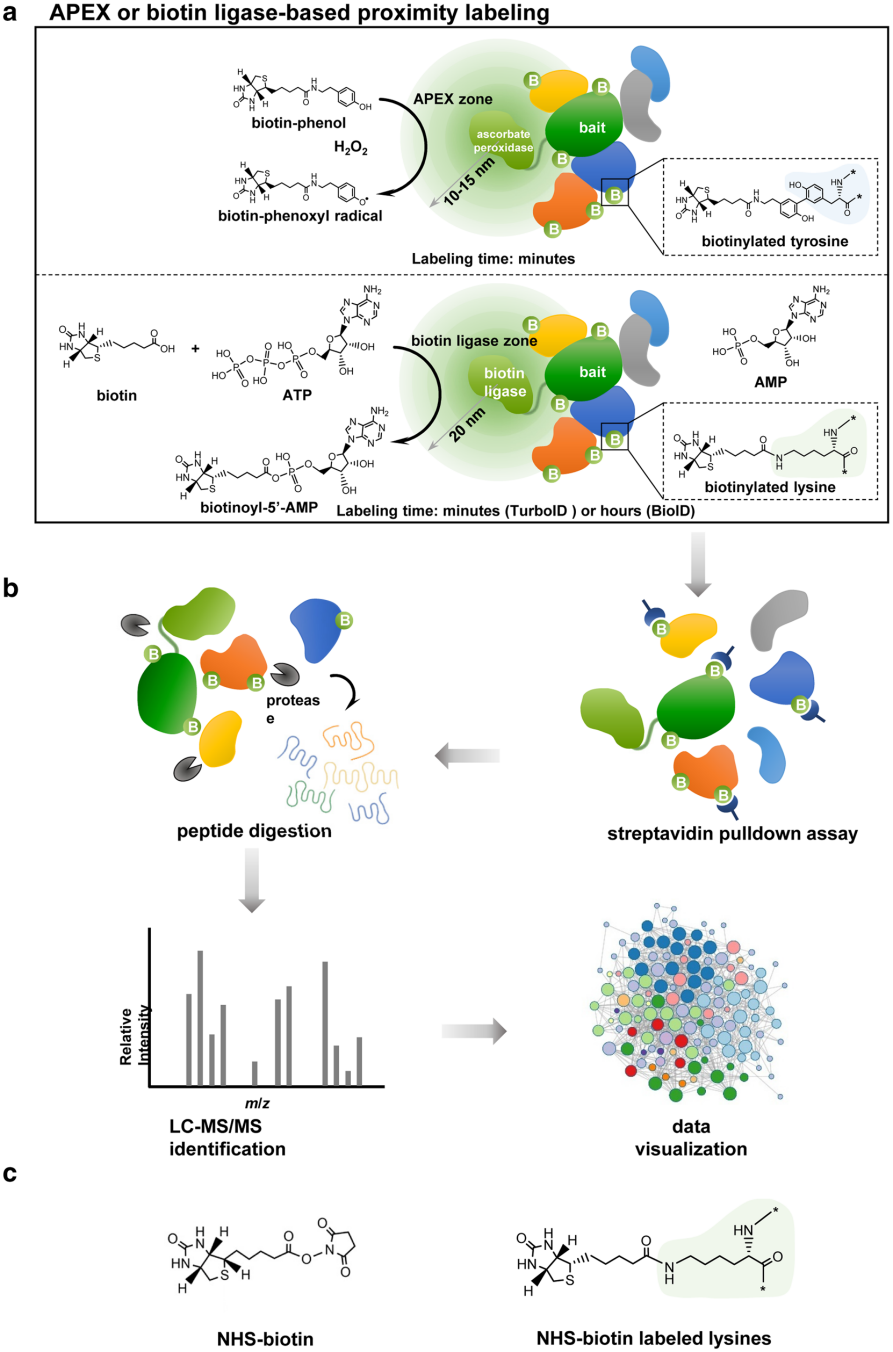
Schematic diagram of enzyme-catalytic proximity labeling approaches. **a** In a standard enzyme-catalytic proximity labeling system, ascorbate peroxidase (e.g., APEX or APEX2) in the presence of H_2_O_2_ catalyzes the one-electron oxidation of biotin-phenol into a highly reactive and short-lived biotin-phenoxyl radical, which biotinylates tyrosine predominantly in nearby proteins (upper panel). In contrast biotin ligase (e.g. BioID or TurboID/miniTurbo) catalyzes the synthesis of a biotinoyl-5’-AMP intermediate from biotin and ATP and promiscuously tags lysine in nearby proteins (lower panel). These enzymes can be fused in frame to the bait protein and introduced into living cells. The biotinylation process depends on the localization of the bait protein and occurs in a proximate dependent manner. **b** The biotinylated proteins are first enriched and purified by streptavidin pulldown assay, then further digested into peptides and identified by quantitative LC-MS/MS. Subsequently, a specific data analysis scheme is adopted for data visualization based on the experimental purpose and design. **c** The chemical structure of NHS-biotin (left panel), which can biotinylate lysine in any protein without a requirement for enzymatic catalyzation (right panel)

APEX is a monomeric 28 kDa ascorbate peroxidase that catalyzes the oxidative polymerization and local deposition of diaminobenzidine (DAB) under harsh treatment conditions; DAB can then be stained with the electron-dense OsO4 to generate strong contrast for electron microscopy (EM) imaging in mammalian organelles [33]. In living cells, exogenous biotin-phenol (BP) can be added and catalyzed by APEX in the presence of hydrogen peroxide (H_2_O_2_) to produce biotin-phenoxyl intermediate; biotin-phenoxyl can covalently react with electron-rich amino acids, such as tyrosine, in proteins in the neighborhood [34]. APEX was therefore adopted for PL (Fig. 1a, upper panel) [34–36]. One of the major limitations of APEX is its relatively low cellular activity and sensitivity, which may arise from its sub-optimal folding/stability, poor heme binding, or some combination of these factors [35]. Thus, in order to provide sufficient biotinylated proteins for subsequent MS identification, higher amount of total protein extracts are typically required. APEX2, which has higher catalytic activity and sensitivity, was later developed through direct evolution [35] and has been successfully used to determine interactomes in living cells [37, 38]. Because APEX2 can also directly biotinylate guanosine in RNAs, APEX-PL has been combined with RNA sequencing (APEX-seq) to determine subcellular transcriptomes [19]. Additionally, APEX2 has been tagged to human telomerase RNA to profile its interactome on a one-minute time scale [18]. Apart from the traditional APEX2 substrate biotin-phenol, a clickable substrate, alkyne-phenol (Alk-Ph), was recently shown to improve membrane permeability and enhance labeling efficiency in intact yeast cells, which enables spatially restricted proteome and transcriptome profiling in yeast [39]. These successful applications demonstrate higher spatial and temporal resolution of APEX-based PL, which is especially suitable for detection of dynamic shifts in interactomes. However, the requirement for sufficient biotoxic heme (H_2_O_2_) to confer high APEX activity limits the in vivo application of APEX-based PL in animals.

Horseradish peroxidase (HRP) is a 44 kDa enzymatic tag with even higher activity than APEX2 that can be utilized for both EM and PL to generate a similar labeling pattern as APEX [36, 40]. The four structurally-essential disulfide bonds of HRP cannot form in a reducing environment, which precludes its applications in cytosol, nucleus and mitochondria. However, it is active in the oxidizing environment, such as secretory pathway and cell surface, and has been employed to map the proteomes of synaptic clefts in living neurons via biotinylation with biotin-xx-phenol (BxxP) and H_2_O_2 _[40]. Lately, HRP has been adapted for an antibody-guided, proximity-based labeling approach, Biotinylation by Antibody Recognition (BAR), to label proteins in primary human tissues without prior insertion of a fusion gene [41]. In a BAR system, the POI in a fixed and permeabilized tissue is initially targeted by the primary antibody, a secondary HRP-conjugated antibody is applied to generate free radicals using BP and H_2_O_2_, and the biotinylated proteins are enriched by AP and detected by MS [41]. BAR obviates the issues associated with protein fusion, but requires a highly specific monoclonal antibody that is sensitive to fixation artifacts.

### The BioID and TurboID system

Initially developed and introduced in 2012, BioID (proximity-dependent biotin identification) uses a highly promiscuous mutated form of *Escherichia coli* (*E. coli*) biotin ligase, BirA*, which has become the most commonly used enzyme for PL [9, 42]. BirA* is a 35 kDa biotin protein ligase that catalyzes the synthesis of biotinoyl-5’-AMP (bioAMP) from biotin and ATP. BioAMP reacts with primary amines, predominantly provided by lysine in any protein, to achieve promiscuous protein biotinylation in a time-dependent manner (Fig. 1a, lower panel) [9]. In the cells expressing BirA* fused bait protein, the proximate proteins of the POI can be biotinylated and probed via streptavidin pulldown assay followed by mass spectrometry (Fig. 1a, b). Unlike APEX, the BioID system simply requires a supply of exogenous nontoxic biotin, which permits its in vivo application. Nevertheless, the slow kinetics of BirA*, which usually takes 18–24 h to generate sufficient biotinylated materials for proteomic analysis [9, 43, 44], precludes using it to capture an interactome snapshot within the timescale of minutes or a few hours. In addition, that BirA* has low or even undetectable catalytic activity in worms, flies, or the ER lumen of cultured mammalian cells [32] makes it problematic for in vivo application. Although newer versions of BioID, BioID2 and BASU, have been developed with improved features [17, 45–47], they continue to have slow catalytic kinetics that limits in vivo use. Because the stability and intracellular lifetime of reactive groups are largely associated with their working radius, it is vitally important to know the exact labeling radius of each enzyme. It was estimated that the effective biotinylation radius of BirA* is about 10–15 nm [43, 48], shorter than that of APEX, which is about 20 nm [49].

Using yeast display-based directed evolution, two engineered promiscuous mutants of BirA*, TurboID and miniTurbo, were developed. These enzymes have superb catalytic efficiency and much faster kinetics than BioID or BioID2 [32]. TurboID takes only 10 min to catalyze nearly as many biotinylated proteins as BioID/BioID2/BASU can provide in 18 hours. In addition, miniTurbo is 20% smaller than TurboID, which may minimize disturbance of fusion protein trafficking and function. It is also preferable for precise temporal control of the labeling window due to less background labeling; miniTurbo does not efficiently label proteins unless a sufficient amount of exogenous biotin is added. On the other hand, TurboID is suggested to be preferred for labeling proteins located in the mitochondrial matrix and ER lumen, and for low-abundant proteins, because it generates stronger signals for these purpose than miniTurbo or BioID2 [32].

In order to improve the spatial specificity and versatility of biotinylation in a PL system, split forms of catalytic enzymes, such as split-APEX [50], split-BioID [51, 52] and split-TurboID [53], have been created. In the split PL system, the promiscuous catalytic enzyme is split into two fragments with no activity on their own; the enzymatic activity can be reconstituted when both units are brought together in cells in a controlled way, either by a chemical molecule or through PPI. Combined with functional studies and screens, split PL is a valuable approach to map the proteomes of organelle contact sites or macromolecular complexes. Unlike split-APEX, split-TurboID does not need the addition of cofactors or co-oxidants. Demonstrated by a study targeting to probe the protein composition of endoplasmic reticulum-mitochondria contact sites, split-TurboID requires a shorter labeling time than split-BioID (4 hours versus 16 hours) and identifies a more balanced set of proteomes, while the split-BioID proteome is relatively biased toward ER membrane proteins [53]. Therefore, split-TurboID offers greater biocompatibility and less bias than split-APEX or split-BioID in mapping the composition of intracellular organelle contact sites.

### In vivo application of PL

Although PL tools have been broadly used in investigations of protein interactomes in a wide range of cultured cells, it is only in recent years that this method has been applied in live animals and plants. Transgenic *Drosophila* or mouse lines carrying APEX or HRP-fused constructs have been reported to identify cell-type-specific proteomes [49, 54–56]. Nevertheless, these in vivo strategies require that living tissues must be dissected and/or perfused first, then incubated with BP or BxxP substrate and subsequently with H_2_O_2_ to activate PL biotinylation. The in vivo BioID/TurboID protocol has overcome these drawbacks. Its use in studies of the c-MYC oncoprotein provides an important example. The c-MYC plays a critical role in the initiation and progression of various types of cancer [57, 58]. For a long time, it remained a technical challenge to characterize the interaction proteins of c-MYC because it is an extremely unstable protein tightly bound to chromatin, making it very difficult to isolate MYC-containing protein complexes using traditional biochemical approaches. The BioID-based PL system yielded more than 100 high-confidence protein neighbors of c-MYC in tumor xenografts grown in mice, which has expanded the interactome of this important oncoprotein to a great extent [59]. In another instance, to capture interactomes of synaptic proteins in mouse brain, adeno-associated viral (AAV)-mediated fused synaptic proteins-BirA cortex expression and subcutaneous injection of biotin enabled the identification of a large number of proteins not previously demonstrated at the inhibitory postsynaptic density, providing a molecular prospectus for the deeper understanding of synaptic physiology [30]. A similar approach was used to identify proteome composition of nascent synapses in cortex and hippocampus of early postnatal mice [31]. The more recently developed BioID knock-in mouse model with BioID inserted at the Z-disc of titin, the giant protein determining the elasticity of myofilament, has provided new insights into sarcomere physiology [48, 60, 61]. Since the expression of titin-BioID at a physiological level leads to a significantly smaller amount of biotinylated materials for MS analysis, cryo-fractured tissue powder digested with trypsin served as the input and anti-biotin antibody facilitated retrieval of biotinylated peptides [62, 63]. Mapping of biotinylation sites to sarcomeric structures deepens our knowledge of myofilament dynamics and supports the model that myosin penetrates the Z-disc to dampen contraction. Furthermore, the proteomic investigation of heart and quadriceps muscle at ages extending from neonatal to adult has linked neonatal signaling pathways to the sarcomere [48]. Another BioID knock-in mouse line with BioID2 fused to the endogenous JPH2 coding sequence was developed to profile the cardiac dyad proteome [64]. This BioID2 knock-in strategy leads to expression levels of JPH2-BioID2 fusion protein comparable to that of the endogenous protein, but still reveals novel potential dyadic proteins that were not discovered using JPH2-HA overexpression transgenic mouse line [65]. In summary, generating biotin ligase (e.g. BioID/TurboID) fusion proteins in animal models has great potential to provide new insights into the molecular mechanisms of human disorders.

Despite attempts to establish a BioID system in *Arabidopsis thaliana* and TMV-infected *Nicotiana benthamiana* plants [66, 67], there continue to be major obstacles to the application of BioID in plants. These include their specialized cell walls and cuticle structures, low growth temperature and low endogenous production and cellular storage of biotin [68]. However, TurboID outperforms BioID and BioID2 in plant studies due to its higher catalytic activity and broader working temperature [26], even with POI of low abundance [8]. Of note, when intact plant tissue is used, it is important to include a free biotin-depletion step to reduce the amount of streptavidin beads required, whereas for mammalian cell culture washing the cells several times seems sufficient to remove excessive biotin [8]. In vivo application of TurboID has also been demonstrated in worms and flies [32], and we expect to see it soon in mouse.

### Important considerations for successful in vivo PL experiments

Clearly, in vivo BioID/TurboID-based PL is an attractive tool with great potential to profile proteomes in a variety of biological situations, even for rare POIs or transient PPIs that are normally difficult to capture by standard biochemical methods. To perform successful in vivo PL investigations, certain aspects of experimental design need to be considered because they are critical to ensure reliable and meaningful results.

1. Choosing the PL labeling enzyme (Table 1): As we mentioned before, APEX and HRP are not ideal for in vivo application due to the need for BP substrate and H_2_O_2_. If the goal is to capture highly dynamic processes that prioritize fine temporal control, the APEX or HRP approach can be used with dissected tissues. The latter is preferable for studies focusing on a secretory pathway or cell surface. BioID has been successfully employed in mouse with different POIs [30, 31, 48, 59]. This success boosts confidence that TurboID, which offers superior catalytic activity and much faster kinetics than BioID, will also be applicable to mouse. The weaker catalytic activity of miniTurbo may be a more advantageous choice when the tissue background has a particularly high endogenous biotin level or a restricted labeling time window is selected because of a lower utilization of endogenous biotin.

**Table 1:** The basic properties and features of enzymatic tags developed for enzyme-catalyzed proximity labeling approaches based on APEX and BioID

Enzyme	Enzyme activity	Year	Size (kDa)	Source	Mutations	Features
APEX	Ascorbate peroxidase	2012	28	Pea	K14D, W41F, E112K	applicability for high-resolution EM tagging of mammalian organelles and specific proteins [36]
APEX2	Ascorbate peroxidase	2015	28	Soybean	K14D, W41F, E112K, A134P	more sensitive and active in cells than APEX for both protein imaging by EM and proteomic mapping[35]; APEX-seq for subcellular RNA detection [19]
BioID	Biotin ligase	2012	35	*E. coli*	BirA-R118G	introduced as a useful screening tool for interacting and neighboring proteins in native cellular environment [9]
BioID2	Biotin ligase	2016	27	*A. aeolicus*	R40G	functionally comparable to BioID, but with more-selective targeting, less biotin supplementation requirement, and enhanced labeling efficiency [45]
BASU	Biotin ligase	2018	28	*B. Subtilis*	Amino acids 1–65 deleted, R124G, E323S, G325R	faster kinetics, increased signal-to-noise ratio compared to BioID, enables direct detection of RNA-protein interactions [17]
TurboID	Biotin ligase	2018	35	*E. coli*	Q65P, I87V, R118S, E140K, Q141R, A146Δ, S150G, L151P, V160A, T192A, K194I, M209V, M241T, S263P, I305V	generates detectable biotinylated materials for analysis within minutes; a superior methods for in vivo studies [32]
miniTurbo	Biotin ligase	2018	28	*E. coli*	Amino acids 1–63 deleted, Q65P, I87V, R118S, E140K, Q141R, A146Δ, S150G, L151P, V160A, T192A, K194I, M209V, I305V	suggested to be less stable than TurboID, but with reduced interference with trafficking and function of fusion protein; preferable when a precisely defined labeling time is the priority [32]
2. When choosing a biotin ligase, it should be considered whether the molecular size or the construction (N- or C-terminal tag) of the enzyme would interfere with the normal function of the POI or with correct targeting to the specific subcellular compartment [8, 32]. A fluorophore or antigenic tag of the fusion protein may expedite confirming its expression and subcellular localization.
3. The expression level of POI-APEX or TurboID is another important factor that significantly affects the interactome analysis. Virus-mediated or transgenic overexpression of the PL enzyme-tagged POI may generate PPIs in vivo that are either not physiological or not specific, burdening the secondary validation procedure. This problem may be solved through CRISPR/Cas9-mediated knock-in to tag the PL enzyme to the endogenous POI at a physiological level [48, 64]. If the endogenous level of PL enzyme-POI yields insufficient biotinylated material for subsequent analysis, the procedure for retrieval of biotinylated peptides may require modification, such as by using cryo-fractured tissue powder digested with trypsin as an input and anti-bio tin antibody [62, 63].
4. One considerable limitation of BioID/TurboID is the presence of non-specific background either due to stochastically biotinylated proteins in the same subcellular localization as the bait protein or because of proteins that nonspecifically bind to the streptavidin-coupled beads. It has been reported that a large proportion of proteins captured in the interactome of a POI are common to all samples, as is usual in affinity purification experiments [8, 31]. It is of critical to set appropriate controls to differentiate high-confidence candidates from non-specific background or contaminant proteins. A free form of biotin ligase targeted to the same subcellular compartment and expressed at the same level as the bait protein is necessary as a negative control [4].
5. It is of vital importance to optimize the experimental conditions to determine the biotin concentration, the duration of biotinylation, and the amount of starting materials. Immunohistochemistry and immunoblotting are common and effective monitoring techniques: the expression patterns and subcellular localization of the biotinylated complex can be visualized by immunochemistry; and immunoblotting is able to semi-quantitatively estimate the efficiency of biotinylation and the minimum starting materials that can be detected. It is worth noting that the signal intensity of immunoblotting does not necessarily reflect the quantity of tagged protein because highly biotinylated proteins may amplify the signal by binding streptavidin at multiple binding sites [8]. Additionally, toxicity analyses in flies and worms indicate that TurboID may sequester endogenous biotin and starve cells of biotin. Therefore, exogenous biotin supplementation is not only necessary for BioID but also for the health of experimental animals. One concern about BioID is that the charge loss on primary amino acids occupied by the covalent attachment of biotin might disturb the formation of other secondary modifications, which could in turn affect the biological behavior of both the fusion protein and proximal proteins. The labeling time should be carefully optimized to determine the shortest possible period that will generate sufficient biotinylated materials but still maintain the spatial specificity of the bait protein; a longer than necessary labeling time may lead to toxicity due to the chronic biotinylation of endogenous proteins [32].
6. Sometimes the strong interaction between avidin, streptavidin or neutravidin-coated beads and biotinylated proteins makes it difficult to elute the bound proteins efficiently. Instead of eluting the biotinylated proteins, digesting them on the beads directly with protease, such as trypsin or Lys-C, may increase the peptide yield [69].
7. Two kinds of MS methods are normally used for final identification of target proteins, label-free and labelled quantification [49]. Introduction of stable isotope labels on the digested peptides, such as Tandam Mass Tag (TMT), or isobaric tags for relative and absolute quantification (iTRAQ), enables identical peptides from diverse samples to be distinguished within a mixture by mass spectrometry. Compared to label-free quantification, these labelled quantification techniques can significantly improve quantitative accuracy at the expense of proteome coverage [70]. When comparing markedly diverse samples, researchers must make certain to select the appropriate data normalization method [8]. Similarly, well-designed bioinformatics analysis is critical to identify high-confidence PPIs.
8. Although the interactome acquired through PL is quite reliable, the ultimate proof is in vivo validation of PPI by complimentary methods together with functional readout demonstrating the interaction is significant and meaningful. Immunofluorescent-chemistry combined with super-resolution imaging can be utilized to validate co-localization of a selected protein candidate and POI; co-immunoprecipitation is an established method to validation the interaction. Development of transgenic animal models in which the expression level of interaction candidates is manipulated by CRISPR will allow in-depth morphological and functional studies [30, 31]. In combination with other state-of-art techniques, this approach will allow us to address challenging questions that are previously inaccessible.

## Conclusions and future perspectives

The dynamic and transient nature of PPIs makes it challenging for conventional approaches to provide real-time in vivo information. The development and in vivo application of BioID/TurboID and its sibling PL method APEX/APEX-seq furnish a powerful toolbox for illustrating critical PPIs and protein-RNA interactions with subcellular resolution. Capitalizing on the high catalytic activity and temporal resolution, TurboID and miniTurbo are likely to be widely employed in mouse in vivo interactome studies. Split forms of the PL method will be more appropriate for studies requiring higher spatial specificity, especially for proteomic analysis of organelle contact sites or macromolecular complexes. In addition to providing interactome profiles using APEX- or BioID-PL techniques, universal protein biotinylation by *N*-hydroxysuccinimidobiotin (NHS-biotin) (Fig. 1c) has recently been adapted to profile the proteome of retinal ganglion cells and transportomes along the visual pathway in adult rats [71]. Another special proteome, nascent proteome, can be determined in mouse in vivo by expressing a mutant methionyl-tRNA synthetase (MetRS L274G), which allows metabolic labeling of newly-synthesized proteins with the non-canonical amino acid azidonorleucine [72]. We expect additional novel or upgraded enzyme tags to be identified in the near future. Combining these approaches will be particularly beneficial for sensitive detection of highly diverse proteomes during a defined time window in a specific cell type in vivo.

## Data Availability

Not applicable.

## Abbreviations

PL: Proximity labeling
PPIs: Protein–protein interactions
AP-MS: Affinity purification-mass spectrometry
BRET: Bioluminescence resonance emission transfer
POI: Protein of interest
LC–MS/MS: Liquid chromatography coupled to mass spectrometry
APEX: Ascorbate peroxidase
EM: Electron microscopy
DAB: Diaminobenzidine
BP: Biotin-phenol
H_2_O_2_: Hydrogen peroxide
HRP: Horseradish peroxidase
BxxP: Biotin-xx-phenol
BAR: Biotinylation by antibody recognition
BioID: Proximity-dependent biotin identification
bioAMP: Biotinoyl-5′-AMP
AAV: Adeno-associated viral
TMT: Tandam mass tag
iTRAQ: Isobaric tags for relative and absolute quantification
NHS-biotin: *N*-Hydroxysuccinimidobiotin
MetRS L274G: Mutant methionyl-tRNA synthetase
